# Modified body mass index as a novel prognostic indicator of in-hospital mortality after off-pump coronary artery bypass grafting: A nationwide multicenter cohort study

**DOI:** 10.1016/j.ijcha.2025.101823

**Published:** 2025-10-15

**Authors:** Shipan Wang, Yilin Li, Hao Han, Tianxu Han, Zhiran Yang, Youjin Li, Haiping Yang, Hongli Li, Gang Liu, Minjia Zhu, Jian Huang, Qingwu Zhao, Jihong Liu, Haibin Li, Shuaitong Zhang, Yuan Xue, Hongjia Zhang, Haiyang Li

**Affiliations:** a Department of Cardiac Surgery, Beijing Anzhen Hospital, Capital Medical University, Beijing, China; b Beijing Institute of Heart, Lung and Blood Vessel Diseases, Beijing, China; c Department of Cardiac Macrovascular Surgery, People’s Hospital of Ningxia Hui Autonomous Region Affiliated to Ningxia Medical University, Yinchuan, Ningxia, China; d Department of Cardiac Surgery, Beijing LUHE Hospital, Capital Medical University, Beijing, China; e Department of Cardiac Surgery, Xuanwu Hospital of Capital Medical University, Beijing, China; f Heart Centre of The First Hospital of Hebei Medical University, Shijiazhuang, Hebei, China; g Institute of Disaster and Emergency Medicine, Tianjin University, Tianjin, China; h Department of Cardiovascular Surgery of the First Affiliated Hospital of Xiamen University, Xiamen, Fujian, China; i Department of Cardiothoracic Surgery, Guangdong Provincial Hospital of Traditional Chinese Medicine Affiliated to Guangzhou University of Chinese Medicine, Guangzhou, Guangdong, China; j Department of Cardiovascular Surgery of the Affiliated Zhongshan Hospital of Dalian University, Dalian, Liaoning, China; k Department of Cardiac Surgery, Beijing Chaoyang Hospital, Capital Medical University, Beijing, China; l School of Medical Technology, Beijing Institute of Technology, Beijing, China

**Keywords:** Modified body mass index, Malnutrition, Risk factor, Off-pump coronary artery bypass grafting, Perioperative complications

## Abstract

**Background:**

Malnutrition is a prevalent yet underrecognized comorbidity in coronary artery bypass grafting (CABG) patients. Modified BMI (mBMI), defined as albumin × BMI, has been proposed as a more accurate indicator of nutritional risk. This study examined the association between preoperative mBMI and in-hospital outcomes following Off-pump CABG (OPCABG).

**Methods:**

This retrospective cohort study included patients with coronary artery disease who underwent isolated OPCABG at eight cardiac centers. Patients were stratified into tertiles based on preoperative mBMI values. The primary endpoint was in-hospital mortality. Logistic regression and restricted cubic spline models were used to evaluate the association between mBMI and outcomes. Subgroup analyses were performed based on clinical characteristics including sex, age, renal function, and comorbidity burden.

**Results:**

Among 6,667 patients, the lowest mBMI tertile had higher in-hospital mortality (2.52 % vs 1.53 % vs 1.17 %), cerebral infarction (2.48 % vs 2.34 % vs 1.39 %), and dialysis rates (1.04 % vs 0.54 % vs 0.36 %) compared to higher tertiles (all P < 0.05). Restricted cubic spline analysis revealed an inverse association between mBMI and the odds of in-hospital mortality, with the lowest risk observed around mBMI 1300–1350. Subgroup analyses demonstrated this relationship was particularly significant among females, patients with eGFR ≤ 90, and those with ASA score > 3 (P for association = 0.023, 0.013, and 0.049, respectively).

**Conclusion:**

Lower mBMI is associated with an increased risk of in-hospital mortality and multiple adverse outcomes in patients undergoing OPCABG, particularly among females and those with renal impairment or systemic comorbidities.

**Trial registration:** Chinese Clinical Trial Registry: Chictr2400085741.

## Highlights

• Clinically accessible mBMI was used to assess preoperative nutritional status.
• Lower mBMI was associated with higher in-hospital mortality risk.
• The association was stronger in women, CKD, and severely comorbid patients.
• Preoperative mBMI helps identify high-risk patients with malnutrition.
• Targeted nutrition may improve outcomes in malnourished OPCABG patients.

## Graphical abstract

**Figure:**
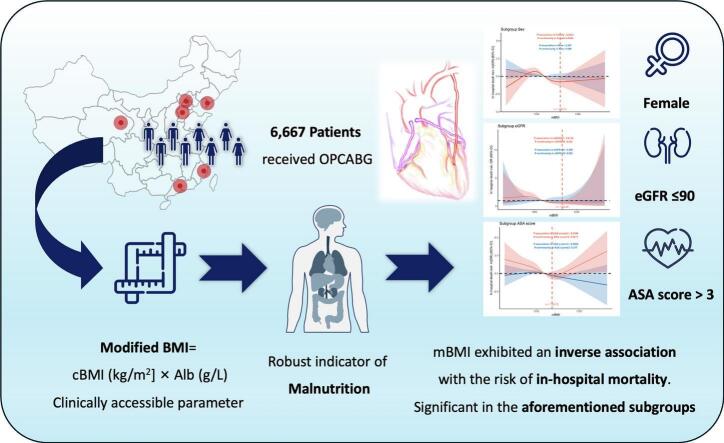

## 1 Introduction

Coronary artery bypass grafting (CABG) is the optimal revascularization strategy for patients with multivessel coronary artery disease [1]. Malnutrition is a common yet frequently underrecognized comorbidity in this population. Epidemiological data indicate that malnutrition affects approximately 9 %–40 % of patients with acute coronary syndrome [2], and up to 40 % of those undergoing cardiac surgery [3]. Most prior research on malnutrition has primarily focused on patients with gastrointestinal diseases and malignant tumors [4], and has revealed a significant association between malnutrition and poor postoperative and intensive care unit (ICU) outcomes [5,6].

The 2021 European Guidelines on Cardiovascular Disease Prevention identified malnutrition as a modifiable risk factor. However, due to the current lack of robust evidence supporting specific therapeutic strategies, malnutrition is often overlooked in routine clinical practice [7]. In cardiovascular populations, malnutrition often coexists with obesity, a phenomenon contributing to the so-called “obesity paradox.” [8,9] As a result, established anthropometric indices such as conventional body mass index (cBMI) may be inadequate for accurately assessing the nutritional status of these patients [10,11]. Although several recent studies and *meta*-analyses have explored the impact of malnutrition on patients with cardiovascular disease, the assessment indicators have varied considerably, and targeted investigations focusing on coronary artery surgery cohorts remain limited [12,13].

The modified body mass index (mBMI)—calculated as the product of serum albumin and cBMI—has gained attention for its simplicity and clinical applicability. Recent studies have employed mBMI to assess malnutrition in cardiac surgery patients and have demonstrated its association with increased risks of mortality and other adverse outcomes [6]. Therefore, this study aims to evaluate the preoperative nutritional status of patients with coronary artery disease using mBMI and to examine its association with in-hospital mortality and other adverse outcomes following off-pump coronary artery bypass grafting (OPCABG).

## 2 Methods

### 2.1 Study cohort

**Fig. 1:**
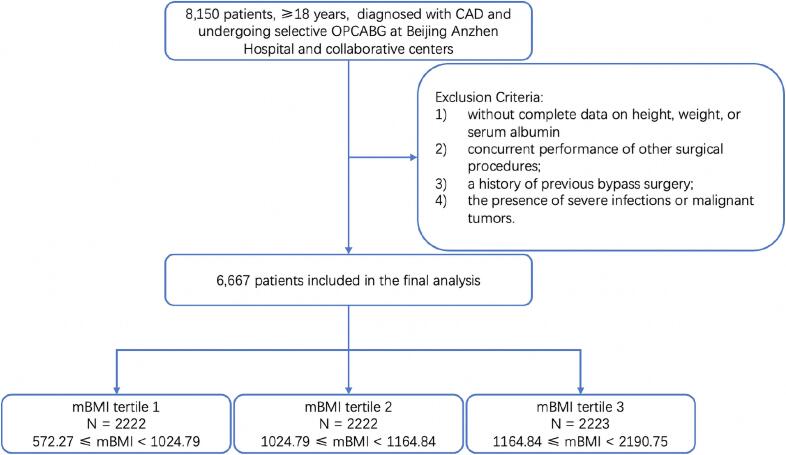
The flow diagram of the cohort design.

This multicenter, retrospective, observational cohort study included 8,150 patients diagnosed with coronary artery disease and who underwent surgical revascularization at one of eight clinical centers. After applying the exclusion criteria: 1) patients without complete data on height, weight, or serum albumin; 2) those undergoing concurrent surgeries; 3) those with a history of CABG; and 4) those with severe infections or malignant tumors, a total of 6,667 patients were included in the final analysis. These patients were evenly divided into three tertiles based on their mBMI values. The study’s cohort design is illustrated in Fig. 1.

This study was conducted in compliance with the Declaration of Helsinki and received approval, including a waiver of informed consent, from the Ethics Review Committee of Beijing Anzhen Hospital, Capital Medical University (approval number: KS2023090). It is based on a registered clinical trial titled “The Preoperative Evaluation of Perioperative Complications and Prognosis Study of Coronary Artery Bypass Graft” (Registration number: Chictr2400085741). Furthermore, clinical data were obtained through a comprehensive review of electronic medical records.

### 2.2 Data collection and definitions

The demographic data of the patients, including age, gender, and smoking status, were recorded upon admission. The medical history, including conditions such as hypertension, diabetes, and prior myocardial infarction, was self-reported and later verified through clinical examinations. Imaging studies were performed and documented following international standards, while laboratory tests were conducted using standardized and certified equipment. Carotid stenosis was defined as ≥50 % narrowing at the site of maximal luminal stenosis on preoperative carotid color Doppler ultrasonography.

The mBMI was calculated by multiplying the conventional body mass index (cBMI, kg/m^2^) by the preoperative serum albumin level (g/L). Height and weight were measured at the time of admission, and the serum albumin level was determined from the first blood sample collected after admission.

### 2.3 Surgical techniques

In this study, all patients with coronary artery disease underwent revascularization exclusively via OPCABG. The number of coronary arteries revascularized was determined preoperatively by the operating surgeon based on coronary angiography findings and was appropriately adjusted intraoperatively according to direct visual assessment. The selection of conduit grafts was based on the surgeon’s experience and included the left internal mammary artery (LIMA), saphenous vein (SV), radial artery (RA), and right internal mammary artery (RIMA). All patients underwent isolated CABG and were transferred to the ICU postoperatively for further monitoring and treatment. Intraoperative data were recorded in the anesthesia records maintained by the anesthesiologist, and the operative records were documented by the primary surgeon.

### 2.4 Study outcomes

The primary outcome of this study was in-hospital mortality, defined as death from any cause during the patient’s hospitalization. Secondary outcomes included other perioperative events, such as postoperative cerebral infarction, postoperative pulmonary infection, postoperative myocardial infarction, the use of intra-aortic balloon pump (IABP), postoperative dialysis, length of stay, ventilator use, and the lowest intraoperative systolic blood pressure.

### 2.5 Statistical analysis

Patients were divided into tertiles based on ascending mBMI values to compare differences in baseline characteristics and perioperative parameters. Continuous variables were expressed as mean ± standard deviation for normally distributed data, or as median with interquartile range for non-normally distributed data and compared using one-way analysis of variance (ANOVA) or the Kruskal–Wallis H test, as appropriate. Categorical variables were presented as absolute numbers and frequencies, and comparisons were performed using the Pearson chi-square test or Fisher’s exact test, as appropriate. We performed univariable logistic regression analysis on the primary outcome to identify additional factors associated with its incidence. The results were expressed as odds ratios (ORs), and variables with P < 0.05 were selected as the basis for subsequent subgroup analyses. Due to the relatively large numerical values of mBMI, which caused some ORs to approximate 1, we further applied a natural logarithmic transformation of mBMI for double-charge analysis. In addition, we evaluated the association of mBMI with the occurrence of other perioperative events using univariable and multivariable regression (logistic for binary outcomes and linear for continuous outcomes). Multivariable analyses adopted a three-step adjustment strategy: Model 1 adjusted for age and sex; Model 2 additionally included prior ischemic stroke, myocardial infarction, and atrial fibrillation; and Model 3 included all Model 2 covariates plus ASA physical status, left ventricular ejection fraction (LVEF), valvular heart disease, and carotid stenosis. Using Model 3 as the classic reference model for the primary endpoint (in-hospital mortality), we then added one marker at a time—mBMI, conventional BMI (cBMI), or serum albumin (Alb)—to construct receiver operating characteristic (ROC) curves and compare areas under the curve (AUCs), thereby quantifying the incremental discriminative value provided by each metric. To assess potential linear or nonlinear associations between mBMI and in-hospital mortality as well as other perioperative events, we constructed restricted cubic spline models to visualize the ORs or ln(ORs) across the range of mBMI values. We further conducted a subgroup analysis to investigate the impact of malnutrition on the prognosis of patients with different clinical characteristics. Stratification of patients was based on the following variables: sex, age ≥ 70 years, history of cerebral infarction, renal function status (eGFR ≤ 90 mL/min/1.73 m^2^ and serum creatinine ≥ 100 µmol/L), ASA score ≥ 3, and the presence of comorbidities such as valvular heart disease and carotid artery stenosis. All statistical analyses in this study were performed using R (version 4.4.2, R Foundation for Statistical Computing) and Python (version 3.12, Python Software Foundation).

## 3 Result

### 3.1 Baseline characteristics

A total of 6,667 patients with severe coronary artery disease who underwent OPCABG were included in the analysis. Patients in the lowest mBMI tertile were notably older on average than those in the highest tertile (mean age 64.5 ± 8.0 vs. 59.7 ± 9.4 years, P < 0.001) and included a higher proportion of females (28.0 % vs. 20.3 %, P < 0.001). Patients with low mBMI exhibited a greater burden of comorbidities as well, including a higher prevalence of previous cerebral infarction (13.4 % vs. 11.1 %, P = 0.042) and renal insufficiency (1.9 % vs. 0.9 %, P = 0.019). By contrast, patients with higher mBMI more commonly had obesity-related conditions such as hypertension (66.4 % vs. 55.9 %, P < 0.001) and hyperlipidemia (51.9 % vs. 44.9 %, P < 0.001). Furthermore, the low-mBMI group had a higher prevalence of carotid stenosis (7.9 % vs. 6.2 %, P < 0.001) and valvular disease (3.2 % vs. 1.5 %, P < 0.001). These baseline characteristics across mBMI tertiles are detailed in Table 1.

**Table 1:** Baseline characteristics of patients.

**mBMI tertile**	**Tertile 1**	**Tertile 2**	**Tertile 3**	**P-value**
N	2222	2222	2223	
mBMI median	943.17 (IQR: 105.19)	1093.58 (IQR:65.23)	1254.69 (IQR:131.76)	
mBMI range	572.27–1024.79	1024.79–1164.84	1164.85–2190.75	
**Preoperative Characteristics**				
**Demographic Characteristics**				
Age (years)	64.52 ± 8.04	62.78 ± 8.38	59.73 ± 9.38	**<0.001**
Female, n(%)	622 (27.99)	561 (25.25)	451 (20.29)	**<0.001**
BMI	23.03 ± 2.09	25.67 ± 1.77	28.86 ± 2.92	**<0.001**
Alcohol, n(%)	556 (25.02)	550 (24.75)	657 (29.55)	**<0.001**
Smoking, n(%)	904 (40.68)	917 (41.27)	1002 (45.07)	**0.006**
**Medical History**				
Hypertension, n(%)	1241 (55.85)	1320 (59.41)	1476 (66.40)	**<0.001**
Diabetes, n(%)	839 (37.76)	824 (37.08)	822 (36.98)	0.843
Hyperlipemia, n(%)	997 (44.87)	998 (44.91)	1153 (51.87)	**<0.001**
Ischemic stroke, n(%)	297 (13.37)	255 (11.48)	246 (11.07)	**0.042**
Atrial fibrillation, n(%)	17 (25.50)	20 (30.00)	21 (31.50)	0.796
Renal insufficiency, n(%)	42 (1.89)	31 (1.40)	20 (0.90)	**0.019**
**Preoperative Clinical Characteristics**				
**Laboratory tests**				
WBC (*10^9/L)	7.96 ± 2.99	8.37 ± 3.29	8.48 ± 3.25	**<0.001**
LYM (*10^9/L)	2.04 ± 22.98	1.61 ± 0.76	1.70 ± 0.74	**<0.001**
PLT (*10^9/L)	208.91 ± 64.74	208.13 ± 61.43	212.55 ± 57.89	**0.005**
Hb (g/L)	123.60 ± 19.93	129.19 ± 20.23	134.78 ± 19.04	**<0.001**
eGFR	85.49 ± 18.41	87.43 ± 16.10	88.61 ± 16.71	**<0.001**
CREA (µmol/L)	84.85 ± 80.64	78.60 ± 29.48	81.39 ± 45.20	**<0.001**
UA (µmol/L)	171.05 ± 169.42	179.26 ± 177.48	184.52 ± 184.84	**0.009**
GLU (mmol/L)	6.27 ± 2.65	6.42 ± 2.51	6.49 ± 2.46	**<0.001**
hsTnI (pg/ml)	132.42 ± 1104.35	65.42 ± 367.30	64.83 ± 474.04	**<0.001**
Potassium (mmol/L)	4.01 ± 0.40	3.99 ± 0.35	4.01 ± 0.34	0.091
Alb (g/L)	40.38 ± 3.23	42.86 ± 2.76	44.82 ± 2.92	**<0.001**
APTT (Sec)	31.72 ± 11.36	31.67 ± 9.18	31.39 ± 3.60	0.145
INR	1.02 ± 0.10	1.01 ± 0.09	1.01 ± 0.07	**0.002**
LAC (mmol/L)	1.53 ± 0.66	1.62 ± 0.69	1.62 ± 0.67	**<0.001**
**Imaging Inspections**				
LVEF (%)	58.79 ± 8.97	59.50 ± 8.31	59.77 ± 7.63	**0.032**
Valvular disease	213 (3.19)	151 (2.26)	101 (1.51)	**<0.001**
Carotid stenosis	526 (7.89)	484 (7.26)	412 (6.18)	**<0.001**
**Intra-operative measurements**				
ASA score	3.06 ± 0.33	3.06 ± 0.31	3.04 ± 0.30	**0.018**
Number of target vascular (n)	3.34 ± 0.94	3.36 ± 0.99	3.39 ± 0.99	0.136
LIMA use, n(%)	1235 (55.58)	1360 (61.21)	1483 (66.71)	**<0.001**

Abbreviations: mBMI **=** Modified Body Mass Index; WBC = White Blood Cell; LYM = Lymphocyte; PLT = Platelet; CREA = Creatinine; UA = Uric Acid; GLU = Glucose; hsTnI = High-sensitivity Troponin I**;** Alb = Albumin; APTT = Activated Partial Thromboplastin Time; INR = International Normalized Ratio; LAC = Lactic Acid; LVEF = Left Ventricular Ejection Fraction; ASA score = American Society of Anesthesiologists Score; LIMA = Left Internal Mammary Artery.

P values in bold are < 0.05.

### 3.2 Perioperative outcomes by mBMI tertiles

Perioperative adverse events and clinical outcomes following OPCABG were strongly associated with preoperative mBMI (Table 2). In-hospital mortality increased as mBMI decreased; 2.5 % of patients in the lowest tertile died during the index hospitalization, compared to 1.5 % and 1.2 % in the middle and highest tertiles, respectively (P = 0.002). Consistently, patients in the lowest mBMI tertile required more intensive perioperative support. Red blood cell transfusion occurred more frequently in tertile 1 than tertile 3 (2.0 % in tertile 1 vs 0.9 % in tertile 3, P < 0.001), and the use of intra-aortic balloon pump (IABP) was nearly twice as frequent in this group (5.1 % vs 3.1 %, P = 0.003). Other post-OPCABG adverse events were more common in the low-mBMI group—cerebral infarction (2.5 % vs 2.3 % vs 1.4 %, P = 0.022), dialysis (1.0 % vs 0.5 % vs 0.4 %, P = 0.014), and re-exploration (1.3 % vs 1.3 % vs 0.5 %, P = 0.009). The hospital stay was also longer (14.1 vs 13.7 vs 13.5 days, P < 0.001). No adverse event was more frequent in the high mBMI group.

**Table 2:** Perioperative characteristics of patients.

**mBMI tertile**	**Tertile 1**	**Tertile 2**	**Tertile 3**	**P-value**
N	2222	2222	2223	
mBMI	943.17 (IQR: 105.19)	1093.58 (IQR:65.23)	1254.69 (IQR: 131.76)	
	572.27–1024.79	1024.79–1164.84	1164.85–2190.75	
In-hospital Death, n(%)	56 (2.52)	34 (1.53)	26 (1.17)	**0.002**
Lowest intraoperative systolic blood pressure (mmHg)	93.88 ± 14.30	94.73 ± 13.32	95.69 ± 12.82	**<0.001**
Operation time (hours)	4.16 ± 0.79	4.16 ± 0.76	4.24 ± 0.79	**0.006**
Blood loss volume (ml)	704.11 ± 310.18	721.94 ± 288.42	757.72 ± 324.91	**<0.001**
RBC infusion category	136 (2.04)	84 (1.26)	58 (0.87)	**<0.001**
RBC infusion (U)	0.14 ± 0.60	0.09 ± 0.51	0.06 ± 0.41	**<0.001**
Length of stay (days)	14.07 ± 5.35	13.71 ± 5.38	13.48 ± 5.10	**<0.001**
Ventilator time (hours)	29.34 ± 40.08	28.80 ± 60.24	25.87 ± 33.89	**<0.001**
ICU stay (hours)	35.54 ± 47.55	35.84 ± 65.54	32.78 ± 49.30	**0.005**
Cerebral infarction, n(%)	55 (2.48)	52 (2.34)	31 (1.39)	**0.022**
Pulmonary infection, n(%)	40 (1.80)	37 (1.67)	39 (1.75)	0.941
Tracheotomy, n(%)	13 (0.59)	4 (0.18)	5 (0.22)	**0.036**
Dialysis, n(%)	23 (1.04)	12 (0.54)	8 (0.36)	**0.014**
IABP, n(%)	113 (5.09)	84 (3.78)	69 (3.10)	**0.003**
Secondary thoracotomy, n(%)	29 (1.31)	29 (1.31)	11 (0.49)	**0.009**

Abbreviations: mBMI **=** Modified Body Mass Index; RBC = Red Blood Cell (Count); ICU = Intensive Care Unit; IABP = Intra-Aortic Balloon Pump.

P values in bold are < 0.05.

### 3.3 The predictive value of mBMI for clinical outcomes

In univariate logistic regression (Table 3), due to the large numeric range of mBMI, its odds ratio appeared neutral (OR = 1.00, 95 % CI: 1.00–1.00, P = 0.013). After log transformation, ln(mBMI) demonstrated a significant inverse association with in-hospital mortality after OPCABG (OR = 0.23, 95 % CI: 0.07–0.72, P = 0.013). Other variables significantly associated with in-hospital death included age, female sex, history of cerebral infarction, valvular disease, carotid stenosis, eGFR, and ASA physical status etc. These factors will be further explored in subgroup analyses. In analyses of log-transformed laboratory values (Supplementary Table 2), higher creatinine and glucose were also significantly associated with mortality. Additionally, lower ln(mBMI) was associated with an increased risk of several adverse outcomes, including cerebral infarction (OR = 0.26, P = 0.014), dialysis (OR = 0.06, P = 0.003), and IABP use (OR = 0.25, P = 0.001), as well as longer duration of mechanical ventilation (β = –8.94, P = 0.014) and prolonged postoperative hospital stay (β = –1.86, P < 0.001) (Supplementary Table 3).

**Table 3:** Univariate logistic regression analysis for in-hospital death.

**Variables**	**OR**	**95 % CI**	**P-value**
mBMI	1.00	(1.00, 1.00)	**0.013**
ln(mBMI)	0.23	(0.07, 0.72)	**0.013**
Female	1.84	(1.25, 2.69)	**0.002**
Age	1.05	(1.02, 1.07)	**<0.001**
Ischemic stroke	1.95	(1.23, 3.07)	**0.004**
Hypertension	1.34	(0.91, 2.00)	0.138
Diabetes	1.33	(0.92, 1.92)	0.134
Renal insufficiency	1.91	(0.59, 6.11)	0.278
AF history	5.52	(2.17, 14.08)	**<0.001**
Old myocardial infarction	1.76	(1.12, 2.75)	**0.014**
Alcohol	1.16	(0.77, 1.73)	0.480
Smoking	0.83	(0.57, 1.21)	0.333
BMI	0.93	(0.87, 0.98)	**0.011**
LVEF	0.97	(0.95, 0.99)	**0.007**
Valvular disease	2.18	(1.27, 3.73)	**0.004**
Carotid stenosis	1.89	(1.28, 2.79)	**0.001**
pre-WBC	0.98	(0.92, 1.04)	0.498
pre-LYM	0.99	(0.78, 1.26)	0.946
pre-Hb	0.99	(0.98, 1.00)	0.124
pre-CREA	1.00	(1.00, 1.00)	**<0.001**
pre-eGFR	0.98	(0.97, 0.99)	**<0.001**
pre-hsTnI	1.00	(1.00, 1.00)	0.627
pre-Alb	0.98	(0.93, 1.03)	0.448
ASA score	1.99	(1.22, 3.24)	**0.006**
LIMA use	0.44	(0.30, 0.64)	**<0.001**
Number of target vessels	0.80	(0.67, 0.96)	**0.019**

Abbreviations: mBMI **=** Modified Body Mass Index; WBC = White Blood Cell; LYM = Lymphocyte; CREA = Creatinine; hsTnI = High-sensitivity Troponin I**;** Alb = Albumin; LVEF = Left Ventricular Ejection Fraction; ASA score = American Society of Anesthesiologists Score; LIMA = Left Internal Mammary Artery; OR = odds ratio.

P values in bold are < 0.05.

We further applied multivariate logistic regression to adjust for the significant variables identified in the univariate analysis (Table 4). Three adjustment models were specified: Model 1 adjusted for age and sex; Model 2 additionally included clinical history variables (prior ischemic stroke, myocardial infarction, and atrial fibrillation); and Model 3 adjusted for all covariates. When ln(mBMI) was treated as a continuous variable, its association with in-hospital mortality did not reach statistical significance after multivariate adjustment. However, when ln(mBMI) was categorized into tertiles, the highest tertile was significantly associated with reduced in-hospital mortality across all three models. (Model 1: OR = 0.55, 95 % CI: 0.34–0.88; Model 2: OR = 0.57, 95 % CI: 0.35–0.92; Model 3: OR = 0.58, 95 % CI: 0.36–0.94) To evaluate incremental discrimination, we constructed a classic model and, in turn, added mBMI, cBMI, or Alb and ROC curves. The model augmented with mBMI achieved a marginally higher AUC than the cBMI- or Alb-augmented models, but the differences were not statistically significant (AUC 0.6483 vs 0.6466 vs 0.6451; Supplementary Fig. S1).

**Table 4:** Multivariate logistic regression analysis for in-hospital death.

ln(mBMI)	OR (95 % CI)		
	Model 1	Model 2	Model 3
Tertile1	Reference (1)	Reference (1)	Reference (1)
Tertile2	0.67 (0.44, 1.03)	0.68 (0.44, 1.05)	0.69 (0.45, 1.06)
Tertile3	**0.55 (0.34, 0.88)**	**0.57 (0.35, 0.92)**	**0.58 (0.36, 0.94)**
P for trend	**0.009**	**0.016**	**0.02**

Abbreviations: mBMI **=** Modified Body Mass Index; OR = odds ratio.

P values in bold are < 0.05.

### 3.4 Exposure–response relationship between mBMI and outcomes

**Fig. 2:**
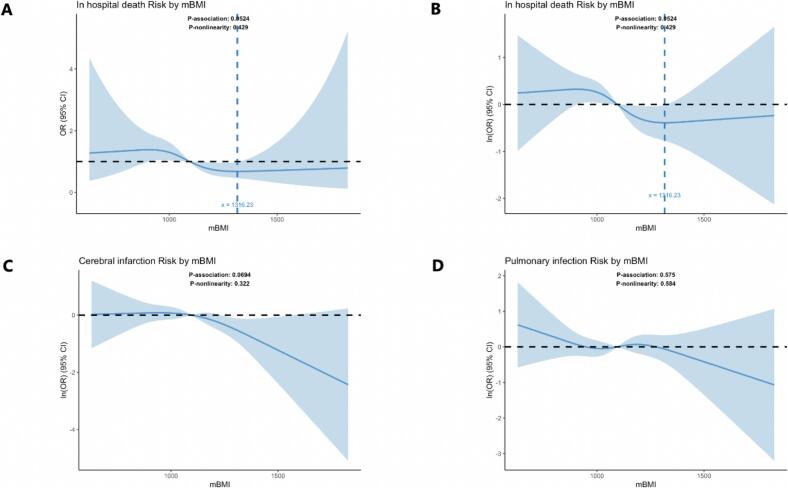
Restricted Cubic Spline Analysis of mBMI and Various Clinical Outcomes. (A) Restricted cubic spline analysis of mBMI and the odds ratio (OR) for in-hospital mortality. (B) Restricted cubic spline analysis of mBMI and the Log-Transformed OR for in-hospital mortality. (C) Restricted cubic spline analysis of mBMI and the OR for postoperative cerebral infarction. (D) Restricted cubic spline analysis of mBMI and the OR for postoperative pulmonary infection.

Fig. 2 displays restricted cubic spline (RCS) curves illustrating the exposure–response relationships between preoperative mBMI and the risk of various outcomes after OPCABG. For in-hospital mortality (Fig. 1, Fig. 1), the spline curve showed that the odds ratio (OR) was lowest at an mBMI of 1316.23 and increased sharply with declining mBMI; however, this trend did not reach statistical significance (P for association = 0.052). Similarly, the ORs for postoperative cerebral infarction (Fig. 1C) and pulmonary infection (Fig. 1D) also demonstrated a decreasing trend with increasing mBMI, but these associations likewise did not reach statistical significance.

### 3.5 Subgroup analyses

**Fig. 3:**
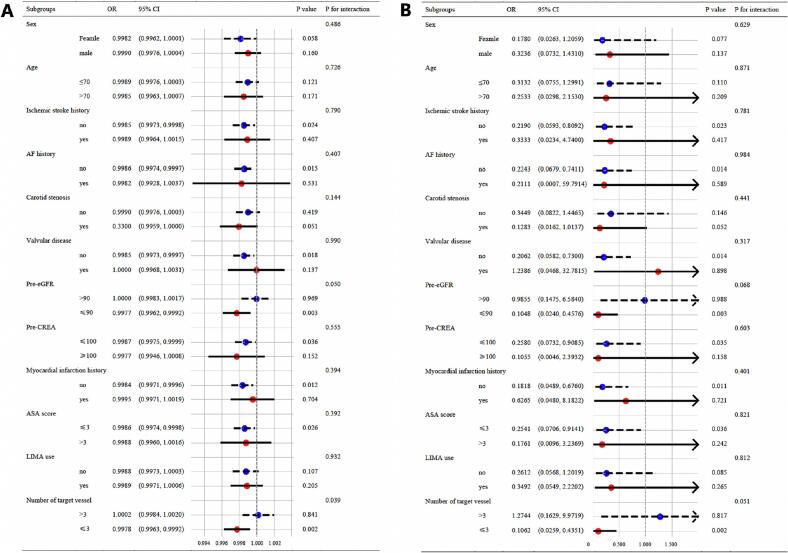
Subgroup Analysis of mBMI and In-Hospital Mortality Risk. (A) Subgroup analysis of mBMI and the risk of in-hospital mortality. (B) Subgroup analysis of ln(mBMI) and the risk of in-hospital mortality. Subgroups include gender, age, history of ischemic stroke, history of atrial fibrillation, carotid artery stenosis, valvular disease, preoperative eGFR < 90, preoperative serum creatinine > 100, history of myocardial infarction, ASA score, use of left internal mammary artery graft, and the number of target vessels.

In subgroup analyses (Fig. 3), lower mBMI was generally associated with higher in-hospital mortality after OPCABG across a range of patient subgroups, but the strength of this association varied according to the clinical characteristics of those coronary artery disease patients. Stratification by preoperative renal function revealed that the inverse mBMI–mortality relationship was significant only among patients with eGFR ≤ 90 mL/min/1.73 m^2^ (OR per unit ln(mBMI) 0.10, 95 % CI 0.02–0.46, P = 0.003), whereas those with eGFR > 90 showed no association (P for interaction = 0.050).

**Fig. 4:**
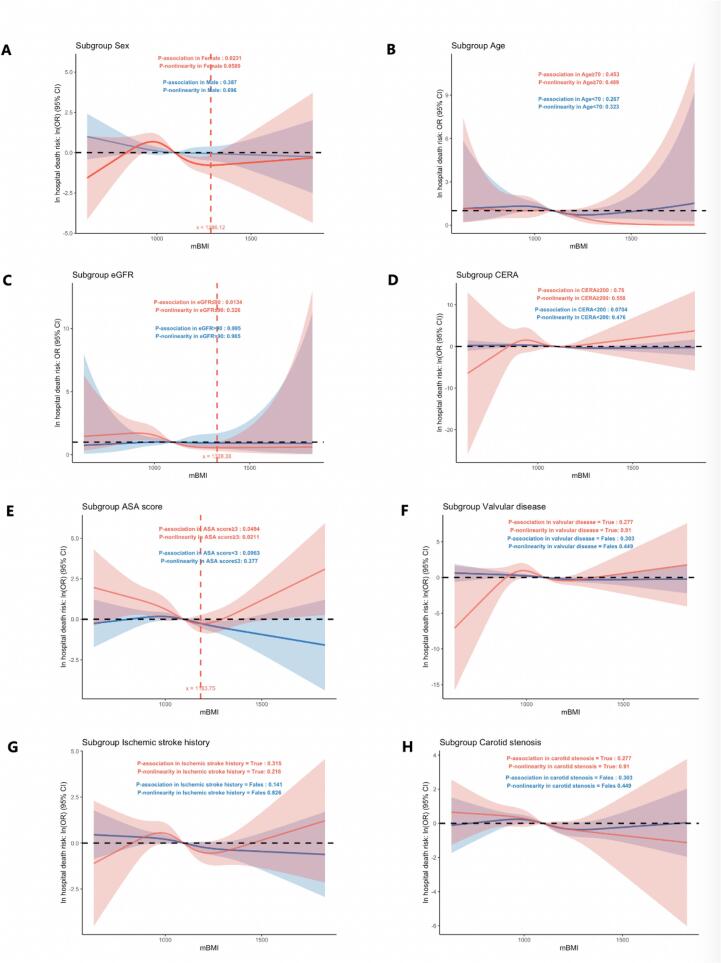
Restricted Cubic Spline Analysis of mBMI and the Odds Ratio (OR) for In-Hospital Mortality Across Subgroups. Subgroup-Specific Associations Presented as Odds Ratios (ORs) and Log-Transformed ORs. (A) Sex subgroup; (B) Age subgroup; (C) eGFR subgroup; (D) Creatinine subgroup; (E) ASA score subgroup; (F) Valvular disease subgroup; (G) History of ischemic stroke subgroup; (H) Carotid stenosis subgroup.

To further explore the prognostic value of preoperative mBMI in specific patient populations, we conducted restricted cubic spline (RCS) analyses across subgroups stratified by age, sex, renal function, and comorbidity status (Fig. 4). While an overall inverse relationship between mBMI and post-OPCABG in-hospital mortality was consistently observed, the strength and shape of this association varied across clinical subgroups. In female patients (Fig. 4A), the exposure–response relationship was statistically significant (P for association = 0.023), with the lowest risk of in-hospital mortality observed at an mBMI of 1286.12, followed by a steep rise in risk as mBMI declined. A similar pattern was noted among those with coronary artery disease comorbid with eGFR ≤ 90 mL/min/1.73 m^2^ (chronic kidney disease stage 1 or beyond) (Fig. 4C), where the association was also significant (P = 0.013), and the lowest mortality risk occurred at an mBMI of 1328.28. Importantly, in patients with an ASA physical status score > 3 before receiving OPCABG (Fig. 4E), indicating severe systemic disease, the relationship between mBMI and in-hospital mortality exhibited a significant U-shaped curve (P for association = 0.049; P for nonlinearity = 0.021). In this subgroup, the lowest mortality risk was observed at an mBMI of 1183.75, with elevated risk at both lower and higher mBMI levels. These findings suggest that the prognostic impact of mBMI may be modified by patient-specific clinical factors, and both malnutrition and excessive adiposity may be detrimental in high-risk surgical populations undergoing CABG.

**Fig. 5:**
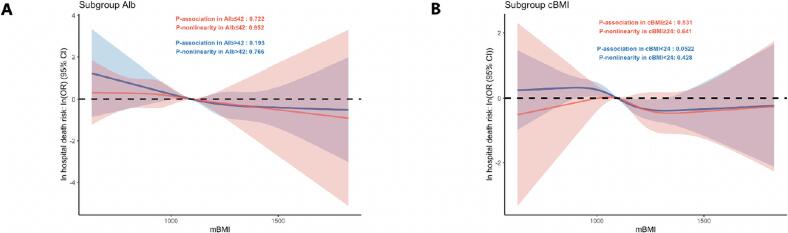
Restricted Cubic Spline Analysis of the Impact of mBMI on In-Hospital Mortality in Groups with High and Low Albumin Levels (A) and High and Low cBMI (B).

Further analyses stratified by serum albumin levels and conventional BMI (cBMI) revealed that the inverse relationship between mBMI and in-hospital mortality remained consistent across these subgroups (Fig. 5). Restricted cubic spline curves demonstrated similarly shaped exposure–response patterns in both high and low albumin as well as high and low cBMI strata, with no significant evidence of effect modification by either variable.

## 4 Discussion

**Fig. 6:**
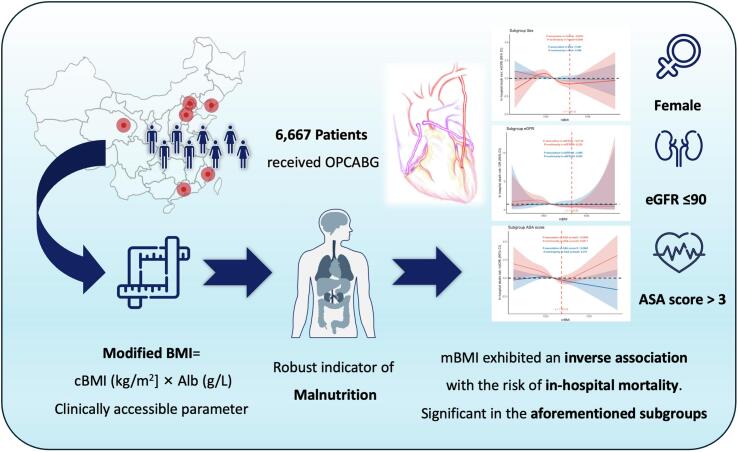

The present study, based on a large cohort of 6,667 patients with coronary artery disease undergoing OPCABG, revealed three key findings: (1) Preoperative demographic characteristics varied significantly across mBMI tertiles: patients with low mBMI were older, had a higher proportion of females, and exhibited a greater burden of vascular and renal comorbidities, whereas those with high mBMI more commonly presented with hypertension, type 2 diabetes mellitus, and lipid metabolism disorders. (2) A lower mBMI was strongly associated with post-OPCABG in-hospital mortality and other adverse perioperative outcomes; these patients also had increased requirements for mechanical life support. (3) An overall inverse exposure–response relationship was observed between mBMI and prognosis, which was more pronounced in high-risk subgroups—patients with chronic kidney disease, females, and those with severe systemic comorbidities. (Fig. 6 Central illustration).

Tertile categorization remained predictive whereas continuous and spline terms did not, for several reasons. First, when excess risk is concentrated in the lower tail, a misspecified global linear or smooth specification averages opposing local slopes and dilutes the effect, a phenomenon exacerbated by sparse events at the extremes. Second, categorization heightens contrast between a low-mBMI risk-enriched group and the referent, improving detectability under finite samples. Taken together, overall, the pattern appears threshold-like on visual inspection, but it is not statistically significant in our spline analyses.

The modified BMI, as compared to cBMI, may provide a more accurate reflection of patients’ preoperative nutritional status. Our study found that although patients with higher mBMI were more likely to have conventional cardiovascular risk factors, postoperative complications following OPCABG were predominantly observed in patients with lower mBMI. This finding addresses the issue of the “obesity paradox” that arises when using cBMI to evaluate the nutritional status of patients undergoing cardiac surgery [8,9]. Studies using cBMI as the exposure variable have reported that patients with low BMI or malnutrition exhibit the highest postoperative mortality and complication rates after CABG, whereas those with moderately elevated BMI tend to have relatively better outcomes [10]. In contrast, other studies have indicated that CABG patients with a conventional BMI exceeding 25 experience higher postoperative mortality than those with BMI values in the normal range (cBMI = 18–25) [11]. This discrepancy may be due to cBMI’s inability to reflect changes in body composition (e.g., hypoalbuminemia), and the fact that conditions such as hypertension and chronic renal insufficiency, which cause water and sodium retention, can elevate cBMI values, thereby obscuring underlying malnutrition [14,15]. Beyond cBMI, prior studies have examined newer indicators of nutritional and physiologic reserve, showing that frailty and sarcopenia predict adverse outcomes after cardiac surgery. Frailty assessment typically requires validated scoring instruments, whereas sarcopenia—though quantifiable on imaging—has not been incorporated into routine CT reporting [16,17]. In comparison, mBMI, through a simple clinical calculation, could more accurately identify patients with elevated cBMI but underlying malnutrition. Moreover, although previous research has suggested that malnutrition may lead to lymphocyte depletion, causing immunosuppression and poor prognosis in patients with acute coronary syndrome [18], our study found no significant differences in lymphocyte counts between the low and high mBMI tertiles, nor any significant association with in-hospital mortality following OPCABG. Thus, the prognostic value of lymphocytes in this patient population requires further investigation.

This study demonstrates a significant association between malnutrition and adverse postoperative outcomes in patients undergoing OPCABG, consistent with previous reports [5,6]. Malnutrition compromises immune defense and reduces metabolic reserves, leading to hypoalbuminemia, which fosters a pro-inflammatory and oxidative stress environment [2,13]. These changes could accelerate the progression of coronary atherosclerosis and, when combined with acute postoperative stress responses, exacerbate cytokine-driven malnutrition, forming a vicious cycle [19,20]. Additionally, malnutrition is often accompanied by endocrine dysregulation, characterized by decreased levels of anabolic hormones (e.g., IGF-1, testosterone) and predominance of catabolic signals (e.g., cortisol, inflammatory cytokines) [21]. This hormonal imbalance promotes muscle wasting and impairs tissue repair capacity, thereby hindering postoperative recovery [[22], [23], [24]]. Moreover, malnutrition-induced loss of muscle mass restricts postoperative mobilization and increases the risk of complications [25,26]. Reduced muscle mass has also been identified as an independent risk factor for major adverse cardiac and cerebrovascular events (MACCE), increasing their incidence by 2.5-fold, while concurrent reductions in myocardial quality and neuroendocrine disturbances further contribute to postoperative cardiac dysfunction [3].

Our subgroup analysis revealed that the association between mBMI and in-hospital mortality was more pronounced among female patients, individuals with chronic kidney disease (CKD, eGFR ≤ 90 mL/min/1.73 m^2^), and those with severe systemic illness (ASA score > 3). Female patients may be particularly vulnerable due to lower baseline muscle mass, smaller body size, and sex-specific hormonal differences (e.g., lower testosterone and IGF-1 levels), which amplify the physiological consequences of malnutrition [20]. In CKD patients, malnutrition is commonly observed due to the combined effects of anorexia, uremic toxins, metabolic acidosis, and chronic inflammation—collectively described as the malnutrition-inflammation complex [27]. A 2022 study noted malnutrition in up to 85 % of CKD patients and identified it as a significant risk factor for cardiovascular events and death [28]. This group lacks sufficient physiological reserve to tolerate surgical stress, and even mild degrees of malnutrition may significantly impact outcomes. Similarly, patients with ASA > 3 represent a population with high comorbidity burden and systemic inflammation, where malnutrition likely interacts synergistically with underlying disease processes to further elevate perioperative risk [20]. In these high-risk groups, mBMI may serve as a sensitive marker of physiologic vulnerability, underscoring the importance of nutritional optimization in these populations before OPCABG.

Conversely, mBMI was not significantly associated with in-hospital mortality in patients aged > 70 years or those with serum creatinine ≥ 200 μmol/L. In elderly patients, prevalent sarcopenia and frailty across BMI categories may reduce the discriminatory value of mBMI, as many with normal BMI may still have low muscle mass (sarcopenic obesity) [29]. The “obesity paradox” in older cardiac surgery patients may also obscure malnutrition-related risks [30]. In patients with severe renal dysfunction, fluid overload, and uniformly high baseline risk limit the usefulness of BMI-based metrics, as body weight may not reflect true nutritional status. Uremia-related complications may further overshadow the impact of mBMI on outcomes [31]. Alternative markers, such as serum albumin or lean body mass, may be more appropriate for risk stratification in these subgroups.

In clinical practice, surgeons can utilize the mBMI to rapidly stratify patients with malnutrition preoperatively and guide targeted management strategies for this high-risk population. Early identification and comprehensive nutritional assessment are imperative, as preoperative strategies—including nutritional optimization and the appropriate postponement of elective surgery—have been shown to significantly reduce postoperative complications. Notably, previous studies indicate that a continuous 10-day preoperative nutritional support regimen in severely malnourished patients can decrease the incidence of postoperative complications by approximately 30 % [32]. Nutritional support should primarily focus on a high-protein diet, supplemented with nutrients such as omega-3 polyunsaturated fatty acids and vitamin C to enhance the patient’s overall nutritional status. When necessary, intravenous albumin supplementation may be considered [[33], [34], [35]]. Additionally, the early initiation of parenteral nutrition within 24 to 48 h during ICU monitoring, coupled with a progressive supplementation of protein and energy postoperatively, further accelerates patient recovery [36,37].

### 4.1 Strengths and limitations

This study has several strengths and limitations. First, this study was based on a 6,667-patient multi-center database containing data from regions across China with varying climates, dietary habits, and disease spectra, enhancing our findings' generalizability and value. However, this study also has some limitations. First, some confounding factors (e.g., the number of procedures involving the aorta during surgery and variations in the duration of general anesthesia) could not be excluded. Moreover, because this retrospective registry had >20 % missingness for NYHA functional class and poor mobility, these variables were not entered into the multivariable models; peripheral arterial disease was represented by carotid stenosis as a proxy for systemic atherosclerotic burden. These data limitations may introduce residual confounding and should be considered when interpreting the results. Then, due to limitations in preoperative assessments and the retrospective design of the study, we were unable to compare mBMI with other widely used nutritional assessment models. Additionally, we reported several subgroup analyses (e.g., stratified by sex, chronic kidney disease, and severe comorbidities). Because multiple testing inflates the type I error rate, these subgroup findings are exploratory and should be interpreted with caution pending replication in independent cohorts. Finally, as a retrospective registry study, current research is subject to potential selection bias and confounding factors, preventing us from establishing a definitive causal relationship between reduced mBMI and in-hospital mortality among OPCABG patients. Future prospective, randomized, multicenter international studies are warranted to compare various malnutrition assessment tools and supportive treatment strategies, aiming to develop more robust perioperative nutritional evaluation and support standards for cardiac surgery patients.

## 5 Conclusion

Categorical (tertile) mBMI was independently associated with higher in-hospital mortality and multiple adverse outcomes after OPCABG. mBMI may serve as a simple yet robust tool to identify high-risk patients, particularly among females and those with renal impairment or systemic comorbidities. These findings underscore the importance of routine nutritional assessment and preoperative optimization strategies to improve postoperative prognosis in coronary surgery.

## Fundings

This study was supported by the Beijing Municipal Science & Technology Commission, Administrative Commission of Zhongguancun Science Park (No. Z221100007422015 & Z241100007724008), Beijing Municipal Administration of Hospitals Clinical Medicine Development of Special Funding Support (No. ZLRL202317), Beijing Natural Science Foundation (No. 7,232,037 & L232030), Beijing Advanced Innovation Centre for Big Data-based Precision Medicine (No. PXM2021_014226_000026) Science and Technology Foundation of Beijing Anzhen Hospital (No. KCGY2023), National Natural Science Foundation of China (No. 82470495) and Beijing Anzhen Hospital High-Level Research Funding (No. 2024AZB2001).

## CRediT authorship contribution statement

**Shipan Wang:** Writing – review & editing, Methodology, Formal analysis, Data curation. **Yilin Li:** Writing – original draft, Methodology, Formal analysis, Data curation. **Hao Han:** Data curation. **Tianxu Han:** Data curation. **Zhiran Yang:** Data curation. **Youjin Li:** Data curation. **Haiping Yang:** Data curation. **Hongli Li:** Data curation. **Gang Liu:** Data curation. **Minjia Zhu:** Data curation. **Jian Huang:** Data curation. **Qingwu Zhao:** Data curation. **Jihong Liu:** Data curation. **Haibin Li:** Methodology. **Shuaitong Zhang:** Methodology. **Yuan Xue:** Writing – review & editing, Supervision, Conceptualization. **Hongjia Zhang:** Project administration, Funding acquisition. **Haiyang Li:** Writing – review & editing, Validation, Supervision, Project administration, Investigation, Funding acquisition, Conceptualization.

## Ethics approval and consent to participate

This research was conducted in accordance with the Declaration of Helsinki, and approval for the waiver of informed consent was granted by the Ethics Review Committee of Beijing Anzhen Hospital, Capital Medical University (approval number: KS2023090).

## Declaration of competing interest

The authors declare that they have no known competing financial interests or personal relationships that could have appeared to influence the work reported in this paper.

## Data Availability

The data presented in this study are available on request from the corresponding author due to ethical restrictions and the protection of patient privacy.
