# Supplementary material for: Modified body mass index as a novel prognostic indicator of in-hospital mortality after off-pump coronary artery bypass grafting: A nationwide multicenter cohort study
Source: Int J Cardiol Heart Vasc. 2025 Oct 15;61:101823. doi: 10.1016/j.ijcha.2025.101823 (PMC12553022; doi:10.1016/j.ijcha.2025.101823)
Supplement: Supplementary Data 1 [file mmc1.docx]

**Supplementary Materials:**

**Supplementary Table S1.** Details of study centers

|  | **Study Implementation Centers** | **Location** | **Roles of the Centers** |
| --- | --- | --- | --- |
| 1 | Beijing Anzhen Hospital, Capital Medical University | Beijing, China, | Principal Study Center |
| 2 | People's Hospital of Ningxia Hui Autonomous Region Affiliated to Ningxia Medical University | Yinchuan, Ningxia, China | Participating Center |
| 3 | Beijing LUHE Hospital, Capital Medical University | Beijing, China | Participating Center |
| 4 | Xuanwu Hospital of Capital Medical University | Beijing, China | Participating Center |
| 5 | The First Hospital of Hebei Medical University | Shijiazhuang, Hebei, China | Participating Center |
| 6 | The First Affiliated Hospital of Xiamen University | Xiamen. Fujian, China | Participating Center |
| 7 | Guangdong Provincial Hospital of Traditional Chinese Medicine Affiliated to Guangzhou University of Chinese Medicine | Guangzhou, Guangdong, China | Participating Center |
| 8 | Affiliated Zhongshan Hospital of Dalian University | Dalian. Liaoning, China. | Participating Center |

**Supplementary Table S2.** Univariate Analysis of the Natural Logarithm (ln) of Preoperative Examination Parameters and the Risk of In-Hospital Mortality.

| **Variables** | **OR** | **95% CI** | **P-value** |
| --- | --- | --- | --- |
| ln WBC | 0.92 | (0.54, 1.57) | 0.761 |
| ln RBC | 0.53 | (0.19, 1.52) | 0.239 |
| ln PLT | 0.77 | (0.42, 1.40) | 0.384 |
| ln Hb | 0.40 | (0.14, 1.11) | 0.078 |
| ln ALT | 0.75 | (0.56, 1.01) | 0.055 |
| ln AST | 0.75 | (0.47, 1.20) | 0.229 |
| ln CERA | 1.74 | (1.07, 2.85) | 0.026 |
| ln GLU | 2.22 | (1.35, 3.67) | **0.002** |
| ln hsTnI | 1.11 | (1.00, 1.24) | 0.052 |
| ln Potassium | 2.53 | (0.33, 19.18) | 0.370 |
| ln Alb | 0.52 | (0.06, 4.57) | 0.555 |
| ln APTT | 1.01 | (0.24, 4.20) | 0.988 |
| ln INR | 0.62 | (0.05, 8.05) | 0.711 |
| ln LAC | 1.14 | (0.72, 1.81) | 0.585 |

**Supplementary Table S3.** Univariate Logistic Regression and Linear Regression Analyses of the Natural Logarithm (ln) of mBMI and the Incidence of Various Clinical Outcomes.

| **Outcomes** | **OR/ß** | **95% CI** | **P value** |
| --- | --- | --- | --- |
| In hospital death | OR = 0.23 | (0.07, 0.73) | **0.013** |
| Cerebral infarction | OR = 0.26 | (0.09, 0.76) | **0.014** |
| Myocardialinfarction | OR = 0.08 | (0.00, 1.33) | 0.078 |
| Pulmonary infection | OR = 0.55 | (0.17, 1.78) | 0.315 |
| IABP | OR = 0.25 | (0.11, 0.55) | **0.001** |
| Dialysis | OR = 0.06 | (0.01, 0.38) | **0.003** |
| Length of stay | ß = -1.86 | (-2.68, -1.04) | **<0.001** |
| Ventilator time | ß = -8.94 | (-16.11, -1.78) | **0.014** |
| Lowest intraoperative systolic blood Pressure | ß = 4.39 | (2.29, 6.48) | **<0.001** |


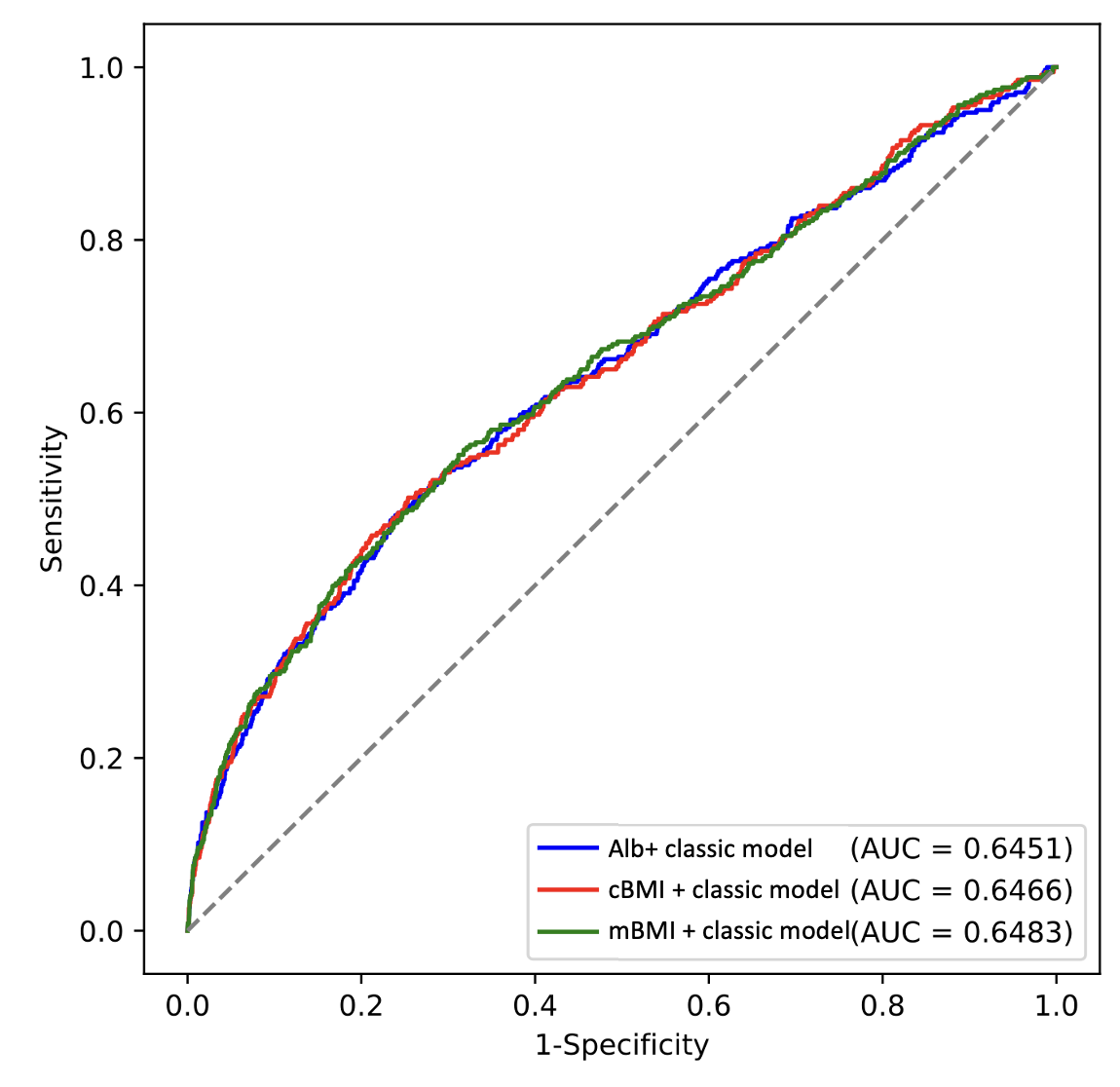


**Supplementary Figure S1**

Receiver operating characteristic (ROC) curves comparing three multivariable logistic regression models for predicting in hospital death.

Alb: Serum Albumin；(m/c)BMI：(Modified/Conventional) Body Mass Index

Classic Model：age, sex, ischemic stroke history, myocardial infarction history, atrial fibrillation history; ASA score, left ventricular ejection fraction (LVEF), valvular heart disease, and carotid stenosis
